# From venom peptides to neurotherapeutics: BmK defensins and short-chain peptides as modulators of ion channels

**DOI:** 10.3389/fphar.2026.1754290

**Published:** 2026-01-29

**Authors:** Yin Dong, Jiajun Wang, Yudan Zhu, Lu Zhao, Yunqing Zeng, Lele Tang, Qian Xiao, Jiwei Cheng, Chao Wang, Jie Tao

**Affiliations:** 1 Joint Laboratory of Nanxiang Branch of Ruijin Hospital-School of Life Sciences, Shanghai University, Shanghai, China; 2 Putuo Hospital, Shanghai University of Traditional Chinese Medicine, Shanghai, China; 3 Shanghai Clinical College, Anhui Medical University, Shanghai, China; 4 Nanxiang Branch of Ruijin Hospital, Shanghai Jiao Tong University School of Medicine, Shanghai, China

**Keywords:** BMK, chloride channels, defensin, neurological disease, potassium channels, short chain toxin, TRP channels

## Abstract

Scorpions, having inhabited the Earth long before the emergence of humans, represent an ancient lineage of arthropods. While often regarded with fear due to their potential to induce severe pain or fatal envenomation, scorpion venoms constitute complex cocktails of bioactive molecules known as toxins. Notably, these toxic components have been repurposed in medical research as valuable sources for therapeutic development. In traditional Chinese medicine (TCM), the venom of *Buthus martensii* Karsch (BmK), commonly referred to as the Chinese scorpion, has been historically employed in the treatment of various neurological disorders, including epilepsy, stroke, glioma, and pain. The principal bioactive constituents of BmK venom are polypeptides that selectively target membrane ion channels. Among these, defensins and short-chain toxins (28–40 amino acids in length) have been identified as key modulators of potassium channels, TRP channels, and chloride channels. These short-chain peptides exhibit several distinct pharmacological advantages, including efficient tissue penetration due to their low molecular mass, remarkable target specificity for particular ion channel isoforms or states, inherently low immunogenicity, and considerable structural versatility that facilitates engineering (e.g., fusion strategies, point mutations) to optimize pharmacokinetics and pharmacodynamics. As such, they represent promising molecular scaffolds for drug design aimed at addressing unmet clinical needs in neurology. We summarize the most advanced drug candidates derived from BmK defensins and short-chain toxins, which exhibit activity against Kv1.3, BK, TRPV1, and other channels implicated in epilepsy, neuroinflammation, glioma, and pain. Structural and functional insights into these peptides reveal mechanisms underlying their target specificity and pharmacological advantages, such as blood–brain barrier penetration and low immunogenicity. This review underscores the originality of BmK peptides as molecular tools and lead compounds for next-generation neurology therapeutics, providing a focused resource for researchers in ion channel pharmacology and peptide-based drug design.

## 1 Introduction

The medicinal use of the BmK scorpion, known in traditional Chinese medicine (TCM) as “Quan Xie” (whole scorpion), dates back to the “Shu Ben Cao” from the Later Shu period (A.D. 934–965) during the Ten Kingdoms of China. Guided by TCM principles such as “Xi Feng Zhi Jing, Gong Du San Jie, Tong Luo Zhi Tong”—which encompass antiepileptic, antitumor, detoxifying, and analgesic effects—BmK has been widely applied in treating neurological and inflammatory conditions, including epilepsy, apoplexy, convulsions, migraine, and tetanus (Xu et al., 2025). In ancient China, “Quan Xie” was typically used in processed forms to reduce toxicity while preserving its active components (Yang F. et al., 2019). It was often combined with other medicinal herbs to enhance efficacy or balance its toxicity (Ling et al., 2019; Wang et al., 2025). The venom of BmK scorpion, regarded as the primary bioactive source, is rich in toxin peptides that modulate the function of various ion channels (Xiao et al., 2022; Wang et al., 2023).

**FIGURE 1:**
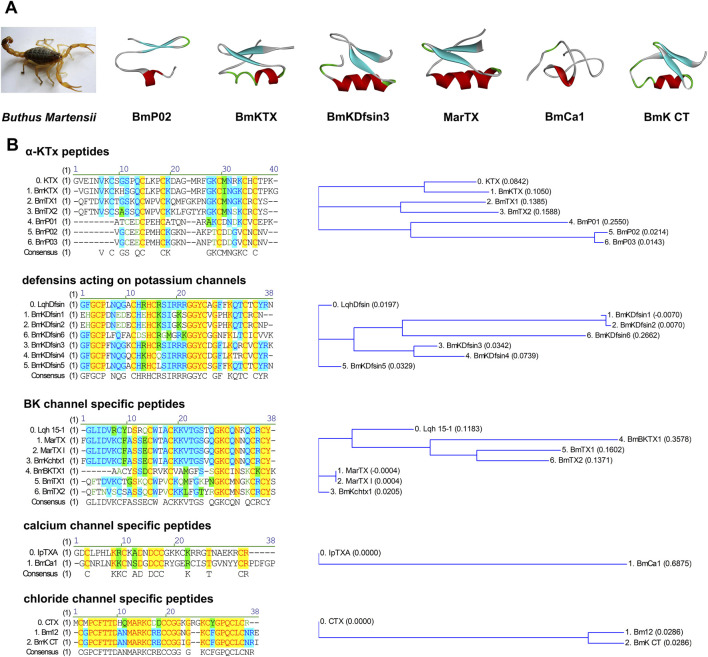
Structural features of short-chain scorpion venom peptides. **(A)** Tertiary Structural Features of Short-Chain Scorpion Venom Peptides. The red regions represent the β-sheet secondary structure, while the blue regions correspond to random coils; the connecting lines (located between structural segments) denote disulfide bonds. **(B)** Primary Structure Sequence Alignment and Phylogenetic Tree Analysis of Short-Chain Scorpion Venom Peptides.

Based on peptide length, BmK toxins are categorized into long-chain and short-chain toxins, including defensins. Long-chain toxins, consisting of 58–76 amino acid residues, primarily target voltage-gated sodium channels (VGSCs). In contrast, short-chain toxins and defensins, comprising 28–40 residues, generally act on K^+^, Cl^−^, or TRP channels (Xin et al., 2025). Based on their structural and functional characteristics, these short-chain potassium channel-targeting peptides are systematically classified into potassium channel scorpion toxins (KTxs), which encompass several subfamilies (Tabakmakher et al., 2019). The α-KTx subfamily is the most abundant and diverse group. Characterized by a conserved cysteine-stabilized α/β (CSαβ) structural motif (where an α-helix and β-sheets are connected by three or four disulfide bridges) and often a functional dyad critical for channel blockade, α-KTx peptides typically range from 23 to 42 amino acids in length (Dauplais et al., 1997; Tabakmakher et al., 2019). Several key drug candidates discussed herein—including martentoxin (MarTX), BmP02, BmKTX, and charybdotoxin (ChTX)—belong to the α-KTx family (Goldstein and Miller, 1993; Banerjee et al., 2013; Selvakumar et al., 2022). Among short-chain toxins, MarTX and BmP02 serve as specific blockers of the BK channel (with β4 subunit) and Kv1.3 channel, respectively (Liu et al., 2020; Liu et al., 2024; Wu W. et al., 2025). Sequence alignment and phylogenetic analysis reveal that MarTX shares low homology with the classical BK channel blocker ChTX from *Leiurus quinquestriatus*, but shows high similarity to Lqh15–1 (ChTX2), another BK channel modulator from the same species (Figure 1).

Additionally, BmCa1 specifically targets calcium channels (Zhijian et al., 2006), while BmP01 potentiates TRPV1 channel activity under acidic conditions (Hakim et al., 2015). BmKCT interacts selectively with chloride channels overexpressed in glioma cells (Du et al., 2018). Notably, BmK defensins, an evolutionarily ancient group of short-chain peptides, not only possess antibacterial and antiviral properties but also specifically inhibit voltage-gated potassium channels such as Kv1.3 (Meng et al., 2016) (Figure 1).

Currently, research on BmK venom in the direction of neurotherapeutic drugs has achieved certain results (Muller et al., 2025). For instance, BmK venom-derived antagonists such as BMK-C205, by targeting the corticotropin-releasing factor receptor, have demonstrated potent antidepressant effects in animal models, contributing to the design of structure-based neuropharmaceuticals (Kim et al., 2023). Additionally, the synthetic peptide SVHRSP derived from BmK venom exhibits pleiotropic neuroprotective functions; in Parkinson’s disease (PD) models, it significantly improves motor dysfunction and neuronal damage by modulating gut microbiota and inhibiting neuroinflammation (Chen et al., 2025). However, their translation into clinical applications continues to face challenges such as limited blood–brain barrier penetration, insufficient *in vivo* stability, and difficulties in scalable production. Addressing these bottlenecks will require interdisciplinary collaboration to overcome existing barriers.

In this review, we systematically summarize the most promising drug candidates derived from BmK venoms, organized according to their biological activities and molecular targets.

## 2 Scorpion venom peptides: Probing and targeting potassium channels in neurological disease

**FIGURE 2:**
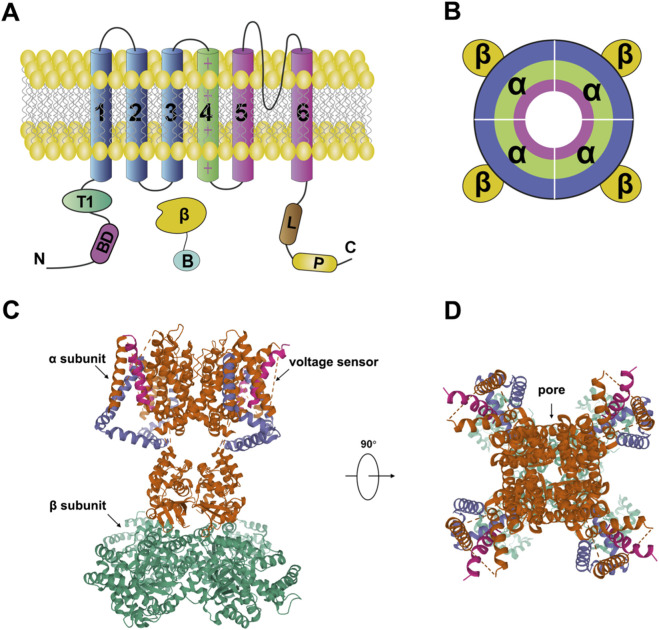
Structure of the voltage-gated potassium channel. **(A,B)** Topology of a voltage-gated potassium channel subunit. Arabic numerals indicate the transmembrane segments (S1–S6) of the potassium channel. The S4 segment, which contains multiple positively charged (“+”) residues, serves as the voltage sensor. The pore region of the channel is formed by segments S5 and S6 from each domain. The intracellular loop between domains III and IV constitutes the inactivation gate (inactivation ball) in voltage-gated sodium channels (Long et al., 2005). **(C,D)** Crystal structure of a voltage-gated potassium channel (PDB: 2A79).

Potassium channels represent the most widespread and diverse class of ion channels in the mammalian, with more than 80 genes have been identified (Perucca and Taglialatela, 2025). Based on their activation mechanisms and the number of transmembrane helices, they are classified into four major families: voltage-gated potassium channels (Kv) (Gutman et al., 2005), calcium-activated potassium channels (KCa) (Zhu et al., 2018), two-transmembrane inward rectifier potassium channels (Kir) (Kobayashi et al., 2006), and two-pore domain potassium channels (K2P) (Kindler et al., 1999). With the exception of K2P channels, which function as dimers, other potassium channels are typically assembled from four homologous or heterologous subunits. These channels often consist of pore-forming α-subunits and auxiliary β-subunits. The α-subunit forms the transmembrane pore that selectively conducts potassium ions, while the β-subunit modulates channel gating and other regulatory functions (Figure 2) (Gutman et al., 2005).

Voltage-gated potassium channels are tetrameric complexes composed of four α-subunits, each approximately 70 kDa in size. In humans, more than 40 Kv channel genes have been identified and categorized into 12 subfamilies (Gutman et al., 2005). Structurally, the Kv1.2 channel (PDB: 2A79) serves as a representative model. Each α-subunit contains six transmembrane segments (S1–S6), with S5–S6 and the intervening P-loop forming the ion conduction pathway. The S4 segment, rich in positively charged residues, acts as the voltage sensor. Upon membrane depolarization, S4 moves outward, pulling the S4–S5 linker and inducing conformational changes in S5 and S6, thereby opening the pore and allowing potassium ions to permeate through the selectivity filter formed by the P-loop (Figure 2) (Long et al., 2005). Physiologically, Kv channels are widely expressed in both excitable and non-excitable cells, where they regulate neuronal excitability (Gonzalez et al., 2012; Martinello et al., 2019), neurotransmitter release (Parekh et al., 2018), immune responses (Chen et al., 2025), and cell migration (Greene and Hoshi, 2017). Given these critical roles, Kv channels are considered promising targets for drug development.

Among them, Kv1.3 has attracted significant attention. It was first identified in human T lymphocytes, where it regulates potassium conductance and provides the driving force for calcium influx, thereby playing a key role in T cell activation, proliferation, and cytokine secretion (Cahalan and Chandy, 2009). Altered Kv1.3 expression has been linked to several autoimmune diseases. For instance, elevated Kv1.3 levels are observed in T cells within inflammatory lesions of multiple sclerosis (MS) patients (Fu et al., 2013). Similarly, in rheumatoid arthritis and type 1 diabetes, activated effector memory T cells (TEM) show upregulated Kv1.3 expression. Blocking Kv1.3 has been shown to suppress TEM activation and mitigate immune-mediated damage, establishing Kv1.3 as a therapeutic target for immunomodulation (Beeton et al., 2006; Wang et al., 2019). ShK, a peptide toxin derived from sea anemone, selectively inhibits Kv1.3 and effectively suppresses TEM-mediated autoimmune responses. A ShK derivative, ShK-186 (Dalazatide), showed no severe or life-threatening adverse effects in Phase I trials. In a Phase Ib trial involving plaque psoriasis patients, subcutaneous administration of Dalazatide twice weekly significantly reduced the Psoriasis Area and Severity Index in 9 out of 10 participants. This agent also shows potential for treating inclusion body myositis, an orphan disease (Tarcha et al., 2017; Wang et al., 2019). The potassium channel blocker OsK (OSK3), isolated from the venom of the scorpion *Orthochirus scrobiculosus*, effectively inhibits Kv1.3 channels (IC_50_ ≈ 503 nM) and Kv1.2 channels (IC_50_ ≈ 331 nM). Due to its potent inhibition of Kv1.2—which may influence central nervous system function—OsK can serve as a valuable tool for studying the structure-activity relationships of potassium channel toxins (Kuzmenkov et al., 2017).

KCa channels are gated by both membrane potential and intracellular calcium levels. Membrane depolarization and elevated cytosolic Ca^2+^ synergistically activate these channels, leading to afterhyperpolarization (AHP) and modulation of cellular excitability. Based on their single-channel conductance, KCa channels are divided into three subtypes: large-conductance (KCa1.1, BK, Slo1, or MaxiK), intermediate-conductance (KCa3.1 or IK), and small-conductance channels (KCa2.1–2.3 or SK).

**FIGURE 3:**
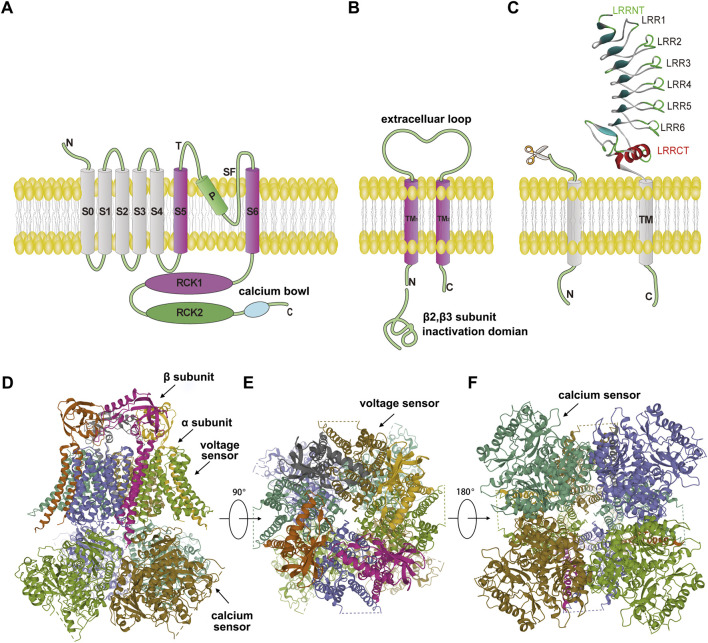
Structure of the large-conductance calcium-activated potassium (BK) channel. **(A)** The α-subunit of the BK channel (PDB: 6V35) is a 7-transmembrane (7TM) protein. The S0 segment is responsible for interaction with auxiliary subunits. Segments S1–S4 constitute the voltage-sensing domain (VSD) of the channel. The intracellular C-terminus contains two RCK (Regulator of Conductance for K^+^) domains (RCK1 and RCK2), which form the calcium-sensing and gating regulation apparatus. Segments S5 and S6 form the outer wall of the pore, and the “P” loop between them lines the selectivity filter. **(B)** The β-subunit is a 2-transmembrane protein. The N-terminus of the β2 and β3 subtypes contains an inactivation gate that mediates rapid inactivation. **(C)** The γ-subunit is a single-pass transmembrane protein. Its extracellular segment comprises conserved domains belonging to the Leucine-Rich Repeat-Containing (LRRC) protein family: LRRNT refers to the Leucine-Rich Repeat N-Terminal Domain, and LRRCT refers to the Leucine-Rich Repeat C-Terminal Domain. **(D–F)** Cryo-electron microscopy (cryo-EM) structure of the BK channel. **(D)** Side view of the cryo-EM structure, showing the tetrameric assembly of the α-subunits. The auxiliary β-subunits associate with the α-subunit tetramer with a 1:1 stoichiometry. **(E)** Top view of the cryo-EM structure, revealing the symmetrical arrangement of the four voltage-sensing domains (VSDs) surrounding the central pore. **(F)** Bottom view of the cryo-EM structure, providing a view of the intracellular tetrameric gating ring formed by the RCK domains, which constitutes the calcium-sensing regulation apparatus.

Among these, BK channels are structurally well-characterized. The α-subunit is encoded by KCNMA1 (slo), while β-subunits are encoded by KCNMB1–4; additionally, γ-subunits are constituted by LRRC family members (Latorre et al., 2017). The human BK channel structure (PDB: 6V35) reveals that the α-subunit contains an extra transmembrane segment (S0) at the N-terminus, which is thought to mediate β-subunit interaction (Tao and MacKinnon, 2019). The voltage-sensing domain is highly similar to that of Kv channels. The pore region, formed by S5, S6, and the P-loop, contains the TVGYG signature sequence that constitutes the potassium selectivity filter. The intracellular C-terminal domain, comprising hydrophobic segments S7–S10, confers calcium sensitivity. A conserved region between S9 and S10 rich in negatively charged residues—known as the “Ca^2+^ bowl”—serves as a high-affinity calcium binding site (Latorre et al., 2017). The RCK1 and RCK2 domains in the gating ring contain at least three additional calcium binding sites and undergo conformational changes upon Ca^2+^ binding, facilitating channel activation (Figure 3) (Latorre et al., 2010; Yuan et al., 2010; Pau et al., 2011).

Four β-subunits (β1–β4) of BK channels have been identified, each showing tissue-specific expression: β1 is prevalent in smooth muscle (Knaus et al., 1994), β2 in adrenal chromaffin cells (Wallner et al., 1999), β3 in testis (Brenner et al., 2000), and β4 in the nervous system (Meera et al., 2000). These smaller (∼20 kDa) β-subunits have two transmembrane segments (TM1 and TM2), with both termini located intracellularly. A conserved disulfide bridge in the extracellular loop stabilizes the structure and facilitates interaction with the α-subunit (Hanner et al., 1998). TM1 and TM2 of β1–β4 interact with the S1 and S2 segments of the α-subunit voltage-sensing domain, modulating channel activation (Figure 3) (Latorre et al., 2017).

BK channels are closely associated with neurological disorders such as epilepsy. For instance, a D434G mutation in the α-subunit accelerates action potential repolarization and increases neuronal firing, leading to generalized epilepsy (Du et al., 2005). In models induced by picrotoxin or pentylenetetrazole (PTZ), pyramidal neurons in the neocortex exhibit enhanced firing frequency and increased BK current density (Shruti et al., 2008). Altered β-subunit function also influences BK activity and seizure susceptibility. The neuron-specific β4 subunit slows channel activation, and β4 knockout mice display symptoms of temporal lobe epilepsy (Liu et al., 2020).

Given the crucial physiological and pathological roles of potassium channels, they are targeted by numerous natural bioactive molecules. From the venom of the scorpion Buthus martensii Karsch (BmK), at least 28 short-chain peptides specifically targeting potassium channels have been isolated, including BmTX1–3, BmKTX, BmBKTX1, BmP01–03, BmP05, BmKK1–4, and MarTX (Zhu et al., 2012). These potassium channel-targeting scorpion toxins are primarily categorized into α-KTx peptides and defensin-like KTx peptides (Rodríguez de la Vega and Possani, 2004). The α-KTx family is the largest, comprising 30 subfamilies. These peptides typically consist of 23–39 amino acids stabilized by three disulfide bonds (CI–CIV, CII–CV, CIII–CVI), except for subfamily six members which contain four. They share a conserved CSαβ structural motif and often interact with the external vestibule of potassium channels *via* N-terminal residues located in the β-sheet region (Rodríguez de la Vega et al., 2003). Pharmacologically, α-KTx peptides act as blockers or inhibitors of BK, SK, and Kv channels. For example, MarTX inhibits neuronal BK channels (Tao et al., 2014; Liu et al., 2020), BmP05 blocks SK channels (Romi-Lebrun et al., 1997b), while BmP01, BmP02, BmP03, BmKTX, and BmTX3 suppress various Kv channels (Zhu et al., 2012). Due to their high specificity and potency, these peptides have become valuable molecular tools for studying ion channel function and demonstrate potential as lead compounds for the development of therapeutics targeting neurological and autoimmune disorders.

### 2.1 Defensins acting on potassium channels

Scorpions, as invertebrates, mainly rely on their innate immune system to combat infectious microorganisms (Charles and Killian, 2015). Defensins are a class of widely expressed cationic antimicrobial peptides (CAPs) that play a crucial role in the innate defense of scorpions (Cociancich et al., 1993). Currently, six defensin genes (BmKDfsin1-6) have been discovered in the whole scorpion. Studies have shown that these defensins are composed of 36–38 amino acid residues and three pairs of disulfide bonds (CI-CIV, CII-CV, CIII-CVI), and they belong to the members of the invertebrate cysteine-stabilized α-helix/β-sheet motif (CSαβ) defensin family (Lang et al., 2017) (Figure 1).

The defensins of the whole scorpion possess certain antibacterial and antiviral properties. BmKDfsin4 has been found to have inhibitory activity against Gram-positive bacteria such as *Staphylococcus aureus*, *Bacillus subtilis*, and *Micrococcus* (Meng et al., 2016). This antibacterial activity can inhibit the growth of bacteria that cause brain infections, thereby reducing the risk of such infections. This potential makes it a promising subject for further research. BmKDfsin4 can also interfere with the replication of Hepatitis B virus DNA and the synthesis of proteins (Zeng et al., 2016). In addition, BmKDfsin3 has been found to inhibit Hepatitis C virus infection in a dose-dependent manner (Cheng et al., 2020).

Furthermore, scorpion defensins have a unique effect on targeting the potassium channel Kv1.3. The Kv1.3 potassium channel is highly expressed on the surface of immune cells such as activated T lymphocytes and is closely related to the occurrence and development of neuroinflammation (DeCoursey et al., 1984). Abnormal activation of the Kv1.3 channel can lead to an exacerbation of the inflammatory response. Scorpion defensins such as BmKDfsin4 can significantly inhibit the current of the Kv1.3 channel at sub micromolar doses (IC_50_ = 510.2 nM), showing the pharmacological characteristics of neurotoxins (Meng et al., 2016), and thus play an anti-neuroinflammatory role, providing a potent molecular tool for studying the mechanisms of neuroinflammation and provides new perspectives for drug target discovery and candidate molecule design in related diseases.

Defensins originated earlier than potassium-channel scorpion venom peptides. While defensins dominate innate immunity, the venom peptides have specialized into tools for predation and defense. Given their extreme structural and electrophysiological similarity (Zhu et al., 2015), it can be inferred that the potassium-channel scorpion venom peptides evolved from defensins. Thus, BmK defensins exemplify the evolutionary ingenuity of scorpion venom peptides, serving dual roles in innate immunity and ion channel modulation. Their ability to selectively inhibit Kv1.3 channels highlights their relevance in neuroinflammation and autoimmune pathology. Building on this foundation, the next sections delve into the more specialized α-KTx peptides, which exhibit even greater target selectivity and therapeutic potential.

### 2.2 α-KTx peptide martentoxin

Martentoxin (MarTX) is a 37-residue α-KTx peptide toxin targeting potassium channels. It exhibits limited sequence similarity (35%–50%) with other scorpion-derived peptides such as BmTX1, BmTX2, BmTX3 and BmKTX from the same species, but shares high homology (75.7%) with Lqh15-1 from the Israeli scorpion *Leiurus quinquestriatus hebraeus* (Romi-Lebrun et al., 1997a; Ji et al., 2003) (Figure 1).

Structurally, MarTX adopts a secondary structure composed of a three-stranded β-sheet and an α-helix. The α-helix spans 10 residues (Ser11–Lys20), while the β-strands comprise Gly2–Asp5, Gln27–Asn30, and Glu33–Cys36. A type I' β-turn, centered at Asn31–Asn32, is formed by residues Cys30–Asn33 (Wang et al., 2006). In solution, the peptide folds into a canonical CSαβ motif, where the α-helix is connected to the β-sheets *via* three disulfide bonds (C8–C29, C14–C34, and C18–C36). The functional face, formed primarily by the β-sheet, differs significantly from classical potassium channel toxins such as ChTX, with surface-exposed aromatic residues suggesting a unique channel interaction mechanism (Wang et al., 2005).

Electrophysiological studies have demonstrated that MarTX at 100 μM partially inhibits the delayed rectifier potassium currents in hippocampal pyramidal neurons, without significantly affecting the transient outward potassium current (Cao et al., 2003). In contrast, at 100 nM, it potently blocks neuronal-type BK channels (composed of α + β4 subunits) and chromaffin cell BK channels, indicating over 1000-fold selectivity for BK channels over other potassium channel types (Ji et al., 2003; Shi et al., 2008).

Structural and functional analyses reveal that MarTX engages the BK channel pore region *via* π–π stacking interactions, including F1–F266 (IV), F9–F266 (II), W15–F266 (I), and Y37–Y294 (IV). Interestingly, MarTX shows no significant activity against BK channels formed solely by the α subunit, suggesting that the β4 subunit is essential for recognition and, together with the α subunit, forms the toxin-binding interface (Liu et al., 2020). Further studies confirmed that MarTX binds the extracellular loop of the β4 subunit (hβ4-loop) at a 4:1 stoichiometry (hβ4-loop: MarTX). Key residues, Glu104, Glu122, Gln124, Lys125, and Glu128 in the hβ4-loop, form a hydrogen-bond network with Asp5, Glu13, Lys20, Ser24, Gln26, Lys28, and Arg35 of MarTX, leading to reduced neuronal firing frequency and prolonged interspike intervals.

Given that BK (α+β4) channel hyperactivity contributes to neuronal hyperexcitability and epileptogenesis, MarTX has been investigated for its antiseizure potential. Intrahippocampal administration of MarTX significantly prolongs seizure latency, reduces seizure duration and incidence, and decreases mortality in pentylenetetrazole-sensitized rats (Liu et al., 2020). It also suppresses c-Fos expression, a marker of neuronal excitation, and exerts neuroprotective effects in the hippocampus.

To enhance blood–brain barrier (BBB) penetration, TAT-fused MarTX variants were engineered. Using a triple-glycine linker, constructs were generated with TAT attached to either the N-terminus (MTX-N-TAT) or C-terminus (MTX-C-TAT) and produced *via* an *E. coli* expression system. Intravenous administration of MTX-C-TAT demonstrated significant antiseizure efficacy, including prolonged seizure latency, reduced severity and frequency of stage 3–5 seizures, suppression of hippocampal hyperexcitability, and neuroprotection. These findings highlight MTX-C-TAT as a promising candidate for intravenous antiepileptic therapy. The successful application of TAT fusion underscores the feasibility of overcoming the BBB delivery challenge for short-chain scorpion peptides, leveraging their small size and engineerability.

Beyond neuronal BK channels, MarTX also modulates other BK channel subtypes. Under high intracellular calcium (micromolar range), it enhances the activity of smooth muscle-type BK (α+β1) channels and glioma BK (gBK) channels. Utilizing MarTX as a molecular probe, studies have revealed a positive correlation between U251 glioma cell proliferation and gBK channel activation (Tao et al., 2011).

Collectively, MarTX emerges as a highly selective BK(α+β4) channel modulator with demonstrated efficacy in preclinical epilepsy models. The successful engineering of BBB-penetrating MarTX variants underscores a tangible pathway for translating venom peptides into practical neurotherapeutics for intractable epilepsy.

### 2.3 α-KTx peptide charybdotoxin

Charybdotoxin (ChTX), a 37-amino acid α-KTx peptide initially isolated from the venom of *Leiurus quinquestriatus* and later identified in related species like *Buthus martensii* Karsch, is a prototypical scorpion venom toxin characterized by a conserved CSαβ structural motif stabilized by three disulfide bonds. Its functional surface, primarily formed by a β-sheet rich in basic residues, enables high-affinity blockade of a subset of potassium channels (Liu et al., 2022). ChTX prominently inhibits voltage-gated Kv1.3 channels (Kd ∼2.6 nM), which are critical for T-cell activation, and Kv1.2 channels (Kd ∼14 nM), thereby modulating neuronal excitability and immune responses (Tajti et al., 2021; Wu Y. et al., 2025). Furthermore, ChTX is a potent nanomolar-scale blocker of the large-conductance Ca^2+^-activated potassium (BK) channel α-subunit, binding directly to its external pore region. Notably, its efficacy against BK channels is allosterically modulated by auxiliary β-subunits; channels incorporating the neuronal β4 subunit exhibit significant resistance to blockade (Liu et al., 2022). The molecular mechanism involves a sophisticated network of interactions, including π-π stacking between toxin aromatic residues (e.g., Trp14, Tyr36) and channel pore phenylalanines, complemented by electrostatic and hydrogen-bonding interactions that secure the toxin within the channel vestibule. This intricate binding underpins ChTX’s value as a molecular probe for delineating potassium channel physiology and its emerging therapeutic potential (Gao and Garcia, 2003). For brain disorders, research has leveraged ChTX to validate BK and Kv1.3 channels as drug targets. Engineered analogs like ChTX-Q18F, designed for enhanced selectivity towards neuronal BK (α+β4) channels, have shown promise in suppressing seizure activity in preclinical epilepsy models by reducing neuronal hyperexcitability (Liu et al., 2022). Beyond epilepsy, ChTX has been instrumental in studying channel roles in ischemic stroke and neuroinflammation, where microglial Kv1.3 inhibition may ameliorate neurodegenerative damage (Beraldo-Neto et al., 2024). Therefore, ChTX serves not only as a foundational tool for probing potassium channel structure and function but also as a template for engineering novel neurotherapeutics. Its subtype-dependent efficacy, particularly with β4-containing BK channels, underscores the importance of subunit composition in drug design. The principal challenges for therapeutic application remain achieving absolute channel subtype selectivity and overcoming the blood-brain barrier, which are being addressed through strategic toxin mutagenesis and advanced delivery systems, highlighting ChTX’s enduring significance in ion channel research and neurotherapeutic development.

### 2.4 α-KTx peptide BmP02

BmP02 is a 28-amino acid α-KTx peptide that targets potassium channels. Sequence analysis reveals that BmP02 shares low homology (35%–50%) with other α-KTx peptides derived from the scorpion *Buthus martensii*, such as BmTX1-3 and BmKTX (Xu et al., 2000). BmP02 adopts the classic CSαβ motif, with its secondary structure consisting of two antiparallel β-strands anchored by three disulfide bonds (C3-C18, C6-C24, and C10-C26) to a single α-helix. The N-terminal α-helix (C10-G13) is relatively short, containing only one helical turn, and its hydrogen bonds can open during conformational changes. The two β-strands include P17-D21 and V23-C26 (Xu et al., 2000). Although BmP02 and the classic SK channel blocker BmP05 are both α-KTx peptide s derived from *Buthus martensii*, their structures differ significantly. BmP02 lacks the R/KXCQ sequence motif on its α-helix, and pharmacological studies have shown that BmP02 has no significant effect on SK channels (Romi-Lebrun et al., 1997b) (Figure 1).

**FIGURE 4:**
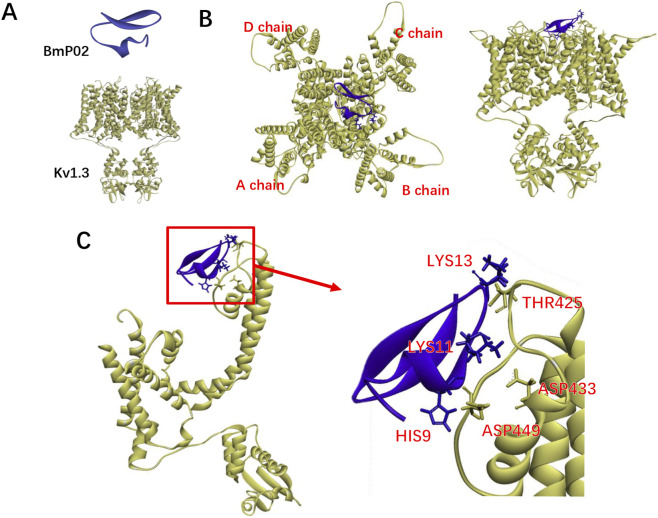
Structural characterization of the BmP02-Kv1.3 binding complex. **(A)** Individual structural components: The upper blue-ribbon diagram represents the short-chain scorpion toxin BmP02 (PDB: 1DU9), and the lower yellow ribbon diagram denotes the homotetrameric Kv1.3 potassium channel (assembled from four identical subunits, consistent with its native architecture) (PDB:7SSX). **(B)** Global binding topology of the BmP02-Kv1.3 complex: The yellow ribbon structure shows the four homologous chains (labeled A, B, C, D) of the Kv1.3 homotetramer; the blue structure corresponds to BmP02. This panel illustrates that BmP02 binds to a conserved site on one of Kv1.3’s chains—notably, all four chains of the homotetramer possess identical binding sites (thus BmP02 can target any chain *via* the same interaction mode). **(C)** Magnified interaction interface of BmP02 and Kv1.3: The left panel displays the local region of the binding site (the red box indicates the magnified area), and the right panel highlights the key interacting residues: The functional residues (HIS9, LYS11, LYS13) of BmP02 (blue) form close contacts with the corresponding residues (THR425, ASP433, ASP449) of Kv1.3 (yellow), which are critical for mediating the specific binding between BmP02 and Kv1.3 (Wu et al., 2016a).

BmP02 has been demonstrated to effectively inhibit Shaker-type potassium channels, particularly showing high affinity for the Kv1.3 channel, with a half-maximal inhibitory concentration (IC∼50∼) of approximately 7 nM (Zhu et al., 2012). Further studies have identified that the three basic amino acids HIS9, LYS11, and LYS13 are critical for BmP02’s interaction with Kv1.3 (Figure 4). When the H451 residue in the pore region of Kv1.3 and the two aspartic acids D421 and D422 in the turret region are mutated, BmP02’s inhibitory effect on the channel is significantly reduced, suggesting that the charge distribution on the outer surface of the Kv1.3 pore plays a crucial role in toxin recognition (Wu et al., 2016a).

Unlike classic α-potassium channel scorpion toxins, BmP02’s functional β-face contains only two β-strands and lacks basic amino acids, indicating that its pharmacological profile may be unique (Wu et al., 2016b). Electrophysiological studies show that BmP02 does not effectively inhibit Kv4.2 currents but significantly delays the channel’s inactivation process, with an EC∼50∼ (concentration for 50% of maximal effect) of approximately 850 nM. Additionally, BmP02 accelerates the recovery of the channel from the inactivated state and slows the deactivation process. When lysine residues on the outer surface of the Kv4.2 pore are mutated, BmP02 no longer delays inactivation but instead exhibits an inhibitory effect, suggesting that BmP02 has two distinct binding modes with potassium channels. Mutations at E4/E5 and D20/D21 in BmP02 do not affect its modulation of wild-type Kv4.2 but still inhibit the lysine-mutated Kv4.2. Conversely, mutations at K11 and K13 render the lysine-mutated Kv4.2 insensitive to the toxin, while the inactivation of wild-type Kv4.2 is still delayed (Wu et al., 2016b).

Thus, BmP02 is a Janus-faced peptide: its basic face interacts with Kv1.3 to block its current, while its acidic face interacts with Kv4.2 to delay inactivation. Such bifunctionality provides insights into channel gating mechanisms and expands the scope for designing context-specific therapeutics. The functional characterization of BmP02 as a highly selective and effective Kv1.3-targeting peptide shows significant research value in developing potential new therapies for human autoimmune diseases.

### 2.5 α-KTx peptide BmKTX

BmKTX is a short-chain scorpion toxin peptide composed of 37 amino acids and three disulfide bonds. It shares ∼87% sequence homology with the classic Kv1.3 blocker kaliotoxin (Figure 1). Pharmacological experiments have shown that BmKTX can completely displace kaliotoxin binding to rat brain synaptosomal membranes at picomolar concentrations (IC_50_ = 0.2 ± 0.07 nM). Similarly, at picomolar concentrations (IC_50_ = ∼0.2 ± 0.01 nM), BmKTX significantly inhibits Kv1.3 channel currents expressed in oocytes (Romi-Lebrun et al., 1997a). These results clearly indicate that BmKTX has the potential to serve as a template for the development of drugs targeting Kv1.3 channel-related autoimmune diseases.

Computer-aided design studies have suggested that mutations at G11R, I28T, and D33H could enhance BmKTX’s selectivity and affinity for Kv1.3, leading to the creation of two gain-of-function mutants: BmKTX-D33H and BmKTX-G11R/I28T/D33H (ADWX-1). Compared to wild-type BmKTX, these mutants exhibit 6-fold and 47-fold higher affinity for Kv1.3, respectively, and their selectivity for Kv1.3 over other potassium channels such as Kv1.1 and Kv1.2 is increased by approximately 10,000-fold and 300-fold (Han et al., 2008; Ye et al., 2016; Li et al., 2019).

At the cellular level, both BmKTX-D33H and ADWX-1 effectively inhibit the secretion of inflammatory cytokines by human and rat memory T cells, mirroring the effects and pharmacological mechanisms of the latest autoimmune disease drug, ShK-186 peptide (Dalazatide) (Li et al., 2019; Wang et al., 2019). At the organismal level, ADWX-1 can alleviate symptoms in experimental autoimmune encephalomyelitis (EAE) models by inhibiting Kv1.3 channels in memory T cells, thereby interfering with IL-2-mediated inflammatory activation pathways, calcium-dependent NF-AT signaling, and PKCθ-triggered NF-κB pathways (Li et al., 2012).

In summary, BmKTX and its analogs demonstrates the value of rational peptide design in developing potent Kv1.3 inhibitors for autoimmune and neuroinflammatory diseases. Its efficacy validates this channel as a therapeutic target and logically leads to the examination of other target families.

## 3 Calcium channel scorpion toxin peptide

As a second messenger in cellular signal transduction, Ca^2+^ plays a vital role in both normal physiological functions and abnormal pathological processes of living organisms. Ca^2+^ enters the cytoplasm through calcium ion channels, transforming electrical signals into chemical signals and mediating the cascade amplification of various cellular signal transductions (Catterall, 2011). These channels are mainly categorized into voltage-gated calcium channels (VGCC), ligand-gated calcium channels (LGCC), and receptor-activated calcium channels (RACC).

VGCC was first identified by Fatt and Katz (1953) and is composed of multiple subunits (Borsotto et al., 1985; Takahashi et al., 1987). In the nervous system, VGCCs serve as the primary channels for calcium influx, maintaining normal neural function by modulating various processes such as neurotransmitter release, synaptic plasticity, and signaling pathways (Jeans et al., 2017; Heck et al., 2019; Horvath et al., 2021). Their dysfunction is associated with a variety of neurological disorders (Nanou and Catterall, 2018). Based on their electrophysiological and pharmacological characteristics, VGCC can be divided into low-voltage-activated (LVA) and high-voltage-activated (HVA) calcium channels. HVA calcium channels consist of α1, β, α2δ, and γ subunits and are further classified into L, N, P, and Q types, with an activation threshold positive voltage of −20 mV in the membrane (Furukawa, 2013). The L-type VGCC family has four members, Cav1.1-1.4, whose α subunits exhibit tissue-specific expression. For example, in the brain, the α1D type is present, which is involved in neuronal firing and calcium signal-dependent gene expression (Ma et al., 2012). In addition to L-type, P/Q-, N-, and R-type VGCC correspond to Cav2.1, Cav2.2, and Cav2.3, respectively. The Cav2 family can interact with SNARE, a key protein in synaptic vesicle transport, to rapidly initiate synaptic transmission (Qian and Noebels, 2001). In contrast, LVA channels, commonly referred to as T-type channels, contain only the α1 subunit and are activated at a membrane voltage of −70 mV. Structurally, they consist of four homologous transmembrane domains, each containing six transmembrane segments (S1-S6), with the channel pore region formed by segments S5 and S6. T-type calcium channels are widely distributed in the thalamus and play an important role in regulating the repetitive firing of rhythmic cells. Compared with other VGCC, this channel can be activated or inactivated more rapidly at a more negative membrane potential (Catterall, 2000). Three subtypes of T-type channels have been identified, namely Cav3.1, Cav3.2, and Cav3.3 (Catterall et al., 2005).

So far, relatively few calcium ion channel peptides have been discovered in scorpions. BmCa1 is a calcium channel toxin-like gene cloned from scorpions using the 5’RACE technique. Based on its cDNA sequence, the peptide precursor consists of 64 amino acid residues, including a 21-amino-acid signal peptide, a 6-amino-acid propeptide, and a 37-amino-acid mature peptide sequence, containing three pairs of disulfide bonds (predicted to be CI-CIV, CII-CV, and CIII-CVI based on the conserved structure of calcium channel toxins). The latest electrophysiological studies have revealed that BmCa1 binds to the S3-S4 extracellular loop of the L-type channel (Cav1.2), delaying channel activation and accelerating inactivation (Zhijian et al., 2006). BmCa1 shares the same cysteine inhibitor knot motif (ICK) with the reported calcium ion channel toxins IpTXA and IpTXi, which is completely different from the CSαβ motif structure of other ion channel peptides in scorpions (Zhijian et al., 2006). In functional studies, live-cell calcium ion imaging has shown that the application of BmCa1 can significantly increase the intracellular calcium ion concentration in primary skeletal muscle cells (Zhijian et al., 2006) (Figure 1).

Intracellular calcium release channels are a type of membrane protein channel located within cells, responsible for regulating the release of calcium ions within the cell. The ryanodine receptor (RyR) is one of the main types of intracellular calcium release channels and is among the largest ion channels known to date. Its opening is modulated by the binding of various endogenous and exogenous ligands (Woll and Van Petegem, 2022). The sodium ion channel peptides BmKAS and BmKAS-1 discovered in scorpions can promote the binding of the RyR receptor in rabbit skeletal muscle cells to the ligand ryanodine, facilitating the release of calcium ions from the endoplasmic reticulum (Ji et al., 1997). The latest research indicates that BmKAS-1 can simultaneously enhance the opening probability of RyR1 and inhibit the activity of the SERCA pump, leading to the continuous depletion of the sarcoplasmic reticulum calcium store. This dual mechanism of action provides a new target for the study of muscle spasm diseases (Julius, 2013).

Receptor-activated calcium channels (RACC) are not only expressed in excitable cells but also widely distributed in non-excitable cells, mediating cellular calcium signaling pathways. RACC can be divided into two types based on their activation mechanisms: store-operated calcium channels (SOC) and receptor-operated calcium channels (ROC). The former is activated by the depletion of calcium stores, while the latter is regulated by intracellular second messengers (Putney et al., 2001; Venkatachalam et al., 2002). The entity of RACC channels had not been identified until the discovery of the transient receptor potential (TRP) channel family, which has opened up a new horizon in this field. This family consists of calcium-permeable, non-selective cation channels that are widely expressed in various tissues and cell types. Studies have shown that some TRP subtypes can mediate calcium influx through mechanisms such as store-operated or receptor-operated pathways, thereby participating in the regulation of intracellular calcium dynamics. The TRP family includes seven subfamilies: TRPML, TRPV, TRPM, TRPC, TRPP, TRPA, and TRPN. Among them, transient receptor potential vanilloid (TRPV) is the most extensively studied TRP channel classification. TRPV1, the first cloned member, is a tetramer composed of 432 amino acids, with six transmembrane regions (S1-S6). The hydrophobic groups of S5 and S6 form the channel pore. Both the N-terminus and C-terminus of TRPV1 are located on the intracellular side. The N-terminus has phosphorylation sites and ankyrin repeats, serving as binding sites for calmodulin and ATP. The C-terminus has a TRP domain, calmodulin, and binding sites for the endogenous TRPV1 inhibitory molecule PIP2 (Moran, 2018). TRPV1 is mainly expressed in small-diameter neurons of the dorsal root ganglia and trigeminal ganglia. This channel can be activated not only by capsaicin but also by endogenous lipids (such as cannabinoids), acidic environments (pH<5.9), and temperatures (>43 °C). TRPV1 was first found to be closely related to the occurrence of heat pain sensitivity in the body. Knocking out the TRPV1 gene can reduce the sensitivity of mice to noxious heat stimuli (Julius, 2013).

BmP01 is the first peptide discovered in scorpions that can act on TRPV1. It is composed of 29 amino acid residues, contains six cysteines, and forms three pairs of disulfide bonds, with the pairing pattern being CI-CIV, CII-CV, and CIII-CVI, representing a typical ICK motif structure. Its three-dimensional spatial structure is composed of one α-helix and two β-sheets. Interestingly, BmP01 was initially found to be a blocker of the potassium channel Kv1.3, with an IC50 of about 270 nM. However, subsequent electrophysiological studies showed that BmP01 can also significantly activate the TRPV1 channel at micromolar doses. At the systemic level, intraplantar injection of BmP01 in mice can induce acute pain-like behavior within 3–5 min, significantly increasing the time mice spend licking their feet. However, this effect is absent in TRPV1 knockout mice (Hakim et al., 2015). In recent years, studies have obtained BmP01-ΔC5 through C-terminal truncation, which significantly reduces its pain-inducing properties (EC_50_ = 1.2 μM), making it a novel non-irritating analgesic lead compound (Hakim et al., 2015).

Scorpion venom-derived calcium channel modulators, though fewer in number, display remarkable mechanistic diversity—from direct pore block of VGCCs to allosteric modulation of TRP channels. Their roles in pain signaling, muscle function, and glioma progression highlight calcium channels as multifaceted targets in neurological and oncological pathologies. This exploration of calcium-active peptides naturally extends to other non-potassium channel targets, such as chloride channels, which contribute to glioma biology and represent an emerging frontier in venom peptide research.

## 4 Chloride channel specific peptide BmKCT

Chloride channels (CLC), as a family of membrane proteins that have not been fully investigated, currently comprise approximately 13 identified members, including CLC-1 to CLC-5 and proton-activated chloride channels (PAC). Previous studies have demonstrated that this channel family plays a critical role in regulating multiple physiological processes, such as cell membrane potential, cellular volume, pH homeostasis, and cell migration, by mediating the transmembrane transport of chloride ions (Jentsch and Pusch, 2018; Yang J. et al., 2019). A recent study has demonstrated that the calcium-activated chloride channel Anoctamin-1 (ANOH-1) mediates the encoding of tactile perception in male *Caenorhabditis elegans* through direct mechanosensation. Human ANO1/TMEM16A protein harbors comparable mechanosensitive characteristics. This work reveals that the chloride channel ANO1 is not solely reliant on Ca^2+^ activation but can also directly transduce mechanical force stimuli (Zou W. et al., 2025).

Analogous to the research status of scorpion-derived calcium channel polypeptides, the discovery of scorpion venom polypeptides targeting chloride channels remains relatively limited (Cohen-Inbar and Zaaroor, 2016). Among these, BmKCT is the most extensively studied representative molecule. Composed of 35 amino acid residues, this polypeptide exhibits a high degree of structural similarity to scorpion venom polypeptides targeting potassium channels, featuring one α-helical domain and two antiparallel β-sheet strands. The specific pairing pattern of its four intramolecular disulfide bonds is CI-CIV, CII-CVI, CIII-CVII, and CV-CVIII (Figure 1). Functional investigations have revealed that BmKCT and its derivative polypeptides can specifically recognize matrix metalloproteinase 2 (MMP-2), which is highly expressed on the surface of glioma cells (Fu et al., 2011), and exert a dose-dependent inhibitory effect on glioma cell proliferation, while showing no significant cytotoxicity toward normal glial cells. These characteristics indicate that BmKCT holds substantial translational medicine value in the development of imaging tracing technologies and targeted drug design for glioma (Fan et al., 2010; Sun et al., 2017; Sun et al., 2019).

## 5 Principles governing Bmk peptide selectivity and affinity

The affinity and selectivity of BmK peptides for ion channels are governed by several fundamental structural and physicochemical principles. First, the number of basic residues in the peptide plays a key role in modulating affinity and selectivity. Studies have found that peptides with fewer basic residues, such as BmK-NSPK, exhibit weaker inhibitory activity against Kv1.1–1.3 and Kv1.6 (inhibiting only 1.2%–5.4% of channel currents at a concentration of 1 μM) (Zuo Z. et al., 2025). This reflects that insufficient basic residues limit their affinity. Secondly, the presence of an amphipathic α-helical structure is closely related to their affinity. Charge and hydrophobicity are primary physicochemical attributes influencing the bioactivity of membrane-active peptides (Rosenfeld et al., 2010; Ong et al., 2014). The amphipathic α-helical structure is a hallmark feature of many BmK peptides. This configuration optimizes the distribution of hydrophobic and charged residues, enabling high-affinity binding to the external architecture of channels and reducing off-target effects. For example, fine-tuning the structure of peptides such as BmKn2-7 can enhance their outer-membrane targeting capability and selectivity (Zeng et al., 2025). Related peptide studies corroborate this point. In the BAK-BH3 peptide, C-terminal extension forms an additional hydrophobic pocket (e.g., the p5 sub-pocket), significantly increasing binding affinity through π-π stacking and hydrophobic contacts (Fogha et al., 2017; Wei et al., 2025).

Furthermore, general peptide-engineering principles indicate that affinity and selectivity can be further enhanced through structural stabilization or by conjugation with advanced delivery platforms. For instance, cyclic peptides can form stable conformations, improving binding affinity, selectivity, and metabolic stability towards targets while reducing toxicity (Wang et al., 2022); nanoparticle encapsulation can improve pharmacokinetics without compromising channel-binding specificity (Keshishian et al., 2025). Similar strategies hold promises for application to BmK peptides to overcome their potential metabolic stability issues (Table 1).

**TABLE 1:** Summary of key characteristics of short-chain scorpion venom peptides. This table summarizes core information of short-chain scorpion venom peptides, categorized by their target ion channels. Columns respectively present: Toxin Name, Target Receptor, Length (aa), Structural Features, Primary Function and Therapeutic Potential.

Toxin name	Target receptor	Length (aa)	Structural features	Primary function	Therapeutic potential
Potassium channels					
Martentoxin	BK(α+β4)	37	3 disulfide bonds; CSαβ motif	Selective blocker of neuronal BK channels	Epilepsy
Charybdotoxin (from Leiurus)	Kv1.3, Kv1.2, BK(α)	37	3 disulfide bonds; CSαβ motif	Potent blockade of Kv1.3/BK channels	Epilepsy, neuroinflammation, stroke research
BmP02	Kv1.3, Kv4.2	28	3 disulfide bonds; CSαβ motif	Blocks Kv1.3; delays Kv4.2 inactivation	Autoimmune diseases
BmKTX	Kv1.3	37	3 disulfide bonds; CSαβ motif	High-affinity Kv1.3 blocker	Autoimmune diseases, neuroinflammation
Calcium channels					
BmCa1	Cav1.2	37	3 disulfide bonds; ICK motif	Delays activation, accelerates inactivation	Muscle spasm, calcium signaling
BmP01	Kv1.3, TRPV1	29	3 disulfide bonds; ICK motif	TRPV1 agonist; Kv1.3 blocker	Pain
Chloride channels					
BmKCT	MMP-2 (on glioma cells)	35	4 disulfide bonds; CSαβ motif	Inhibits glioma proliferation	Glioma

## 6 Prospect

In the treatment of neurological disorders, venom-derived short-chain peptides, such as those from scorpions, demonstrate distinctive advantages in targeted regulation. Their core mechanism is rooted in the evolutionarily refined, high-affinity recognition of specific neural receptors and ion channels, as exemplified by MarTX’s selectivity for BK(α+β4) channels and BmKTX’s potency against Kv1.3. Through precise mimicry of endogenous neuropeptides (e.g., enkephalin, neurotensin), short-chain peptides exhibit selective binding to G protein-coupled receptors (GPCRs) or ion channels (e.g., TRPV, NMDA receptors) (Davenport et al., 2020; Jimenez, 2022; Zong et al., 2022; Tang et al., 2025a), thereby enabling precise modulation of neural signaling pathways. For example, a synthetic peptide modified from the tarantula venom peptide GsMTx4 significantly alleviates mechanical allodynia in rat models by blocking the mechanosensitive ion channel TRPV4, while avoiding side effects such as opioid-like addiction and respiratory depression (Ke et al., 2025); μ-conotoxins (such as μ-KIIIA) can specifically block the voltage-gated sodium channel subtype Nav1.7. After stability optimization through point mutations, their analgesic potency is enhanced and metabolic stability is improved, offering a new strategy for opioid-alternative therapies (Li et al., 2024; Tang et al., 2025b). Mechanistically, such agents circumvent the off-target effects of small-molecule drugs, thereby significantly reducing adverse reactions, such as cognitive impairment and somnolence, induced by conventional neurological pharmaceuticals (Beattie and Sher, 2025). Additionally, the metabolic safety profile of short-chain peptides provides critical clinical utility: their degradation products consist of natural amino acids without organ accumulation toxicity, rendering them suitable for chronic neurological diseases requiring long-term therapy (e.g., Alzheimer’s disease, Parkinson’s disease) (Apostolopoulos et al., 2021). For example, GpTx-1, a peptide derived from tarantula venom, demonstrates potent analgesic effects in various pain models (acute pain, neuropathic pain) without causing motor impairment or tolerance, and its side effects are significantly lower than those of morphine, indicating its potential for development as a novel analgesic drug (Biswas et al., 2017; Chen et al., 2018). Furthermore, with a molecular weight below 5 kDa, their immunogenicity is significantly lower than that of antibody-based drugs, minimizing the risk of intracerebral inflammation (Kyyriäinen et al., 2024). Although most peptides exhibit limited blood-brain barrier (BBB) permeability, certain cell-penetrating peptides (e.g., RGD peptides) can traverse the BBB *via* receptor-mediated endocytosis or passive diffusion (Varnamkhasti et al., 2020; Niu et al., 2021). Combined with modification strategies such as lipidation and pegylation, these approaches further enhance brain-targeted delivery efficiency. The short metabolic half-life of short-chain peptides (ranging from minutes to hours) confers advantages in clinical scenarios requiring rapid dose titration (e.g., epileptic seizure intervention), while the reversibility of side effects upon drug discontinuation aligns with the requirements of dynamic neural regulation (Alé et al., 2014).

Notwithstanding their substantial advantages, the clinical translation of short-chain peptides remains encumbered by multiple challenges: first, poor *in vivo* stability represents a critical limitation as native peptides are highly susceptible to proteolytic degradation, leading to short plasma half-lives, though structural modification strategies such as D-amino acid substitution, cyclization design (e.g., disulfide bond cyclization in octreotide), and N-methylation have been developed to enhance degradation resistance (Yin et al., 2025), while long-acting technologies like fatty acid chain modification (e.g., semaglutide) and microsphere sustained-release formulations (e.g., octreotide LAR) have extended the duration of action to weeks as successful clinical examples; second, low oral bioavailability (typically <2%) restricts administration flexibility, yet research demonstrates that nanocarrier encapsulation (e.g., liposomes, polymer microspheres) can protect peptides from gastric acid degradation—exemplified by the exenatide extended-release microsphere formulation (Bydureon) (Davenport et al., 2020), and combinatorial use of enzyme inhibitors (e.g., DPP-4 inhibitors with GLP-1 analogs) can suppress intestinal peptidase activity to enhance oral absorption efficiency (Davenport et al., 2020); additionally, high costs of large-scale production pose a significant industrialization bottleneck, though automation of solid-phase peptide synthesis (SPPS) *via* continuous-flow reactors has significantly improved production capacity and reduced costs for long-chain peptide synthesis (Jimenez, 2022), while green processes such as molecular distillation and pipeline reaction technologies have minimized waste generation to meet environmental sustainability standards in pharmaceutical manufacturing (Jimenez, 2022).

The future research on short-chain peptides can focus on the following five innovative directions: first, the development of intelligent prodrug systems aims to achieve precise spatiotemporal activation through bioorthogonal reactions. For example, the tetrazine linker (voTz) developed by Sichuan University can trigger the release of active peptides at tumor or inflammatory sites to avoid systemic toxicity (Niu et al., 2021), demonstrating application potential in targeted therapy of brain tumors (e.g., RGD-Dox conjugate drugs) and local intervention of neuroinflammation (Niu et al., 2021). Second, peptoid and multifunctional mimic peptide design avoid enzymatic cleavage sites by relocating peptide bond side chains to nitrogen atoms while preserving targeting ability (Shao et al., 2024). A successful example of this strategy is the GLP-1 analog liraglutide, which extends its half-life to 13 h through fatty acid chain modification (Davenport et al., 2020). Third, peptide-nucleic acid conjugate (PNA/PNAC) technology provides a novel therapeutic paradigm for gene-related neurodegenerative diseases (e.g., Huntington’s disease, spinal muscular atrophy) by conjugating targeting peptides with siRNA or antisense oligonucleotides (Tsylents et al., 2023). Fourth, AI-driven rational design and high-throughput screening can significantly accelerate candidate drug optimization by predicting peptide conformations and receptor binding modes through computational simulation, combined with evaluating neuroregulatory efficacy using brain organoid models (Hongen et al., 2024). Fifth, the development of neuroimmune bidirectional regulatory peptides focuses on modulating microglia and astrocytes (Apostolopoulos et al., 2021). For instance, GsMTx4-derived peptides can simultaneously inhibit mechanical nociceptive transmission and neuroinflammatory pathways, providing new targets for intervening in neuro-immune interactions (Apostolopoulos et al., 2021).

In summary, venom-derived short-chain peptides, characterized by precise target specificity, metabolic safety, and engineerable properties, have established themselves as a transformative frontier in the development of pharmaceuticals for neurological disorders. Future advancements will hinge on the profound integration of technological innovations, interdisciplinary methodologies, and therapeutic paradigms: the convergence of prodrug design with nanocarrier systems will enable spatiotemporally precise activation at pathological sites (Niu et al., 2021); the synergistic application of artificial intelligence (AI)-driven computational modeling and brain organoid platforms will expedite rational drug design workflows (Badai et al., 2020) and bidirectional modulation of neural regulatory and immunomodulatory pathways (e.g., GsMTx4-derived peptides) holds promise for expanding therapeutic horizons (Apostolopoulos et al., 2021). As long-acting formulation technologies, brain-targeted delivery systems, and intelligent prodrug strategies mature, short-chain peptides are poised to transition from symptomatic management to neural repair, fundamentally redefining the therapeutic landscape for refractory neurological conditions such as Parkinson’s disease and Alzheimer’s disease.
